# Use of Plasma Technologies for Antibacterial Surface Properties of Metals

**DOI:** 10.3390/molecules26051418

**Published:** 2021-03-05

**Authors:** Metka Benčina, Matic Resnik, Pia Starič, Ita Junkar

**Affiliations:** Department of Surface Engineering, Jožef Stefan Institute, Jamova 39, SI-1000 Ljubljana, Slovenia; metka.bencina@ijs.si (M.B.); matic.resnik@ijs.si (M.R.); pia.staric@ijs.si (P.S.)

**Keywords:** metal biomaterials, antibacterial properties, plasma treatment

## Abstract

Bacterial infections of medical devices present severe problems connected with long-term antibiotic treatment, implant failure, and high hospital costs. Therefore, there are enormous demands for innovative techniques which would improve the surface properties of implantable materials. Plasma technologies present one of the compelling ways to improve metal’s antibacterial activity; plasma treatment can significantly alter metal surfaces’ physicochemical properties, such as surface chemistry, roughness, wettability, surface charge, and crystallinity, which all play an important role in the biological response of medical materials. Herein, the most common plasma treatment techniques like plasma spraying, plasma immersion ion implantation, plasma vapor deposition, and plasma electrolytic oxidation as well as novel approaches based on gaseous plasma treatment of surfaces are gathered and presented. The latest results of different surface modification approaches and their influence on metals’ antibacterial surface properties are presented and critically discussed. The mechanisms involved in bactericidal effects of plasma-treated surfaces are discussed and novel results of surface modification of metal materials by highly reactive oxygen plasma are presented.

## 1. Introduction

Device-associated infections may cause severe problems connected with long-term antibiotic treatment, implant failure, and even risk for serious complications, potentially fatal for the patient. Implant-associated infections present a tremendous economic burden also due to high hospital costs. Moreover, accompanying antibiotic resistance also poses an increased threat to the public health, as antibiotic-resistant bacteria kill around 33.000 patients in Europe and produce financial costs of about €1.5 billion per year [1]. Bacterial adhesion on implantable medical devices thus presents a serious health concern, as among all possible complications the implant infections occur in 1 to 13 percent of the cases [2]. Commonly applied action against postoperative implant infections include treatment by systemic antibiotics [3]. The problem with such treatment is the difficulty of the antibiotic to achieve high enough concentration through blood circulation at the infection site to act effectively against the bacteria. With systemic administration of antibiotics, there is a greater chance of side effects of such treatments and also a higher risk of increasing antibiotic resistance of bacteria. Combining desired implant material with antibacterial surface properties could solve such clinical problems [4].

Many strategies have been proposed to fabricate antibacterial materials, mainly the development was directed in surface modification. Surface modification techniques can be divided into approaches based on physico-chemical surface modification and coating of surfaces with biocidal agents, e.g., inorganic complexes (containing bactericidal cations) chemically grafted to bulk surfaces [5], while recently also the fabrication of biomimetic surfaces is becoming increasingly popular [6]. The physico-chemical surface modification techniques induce changes in surface chemistry and nanostructure, which influence the surface-cell interactions by disrupting molecular recognition of surface by bacteria and/or physically intervene with the formation of biofilms. Surface modification interferes with bacteria attachment on the surface and the formation of biofilms. Recently, approaches based on biomimetic surfaces were also vastly studied. These strategies deal with nanostructuring of the surface and may offer prevention of bacterial adhesion and simultaneously promote desired cell lines [7]. The another type of surface modification methods are biocide-based strategies, where antibacterial agents are incorporated in biomaterial coating and released into the surrounding environment by predefined course of action, or the antibacterial agent is immobilized on the surface of the biomaterial, which either prevents bacterial attachment or inactivates bacterial cells when in contact with the surface [8,9].

Surface coating techniques such as ion implantation [10], electrochemical anodization [11], ion exchange [12,13], sol-gel techniques [14], plasma spraying [15,16], and incorporation of metal ions such as silver, copper, or zinc have been vastly studied in past decade [13,17,18]. These approaches have been shown to improve the materials’ antibacterial properties of the materials, however many of them are complex and have several limitations. Specifically, antibiotic-loaded coatings on metal surfaces are associated with the evolvement of multi-drug resistance of bacteria [18]. With the growing field in nanotechnologies, much of the recent research has been devoted to the development of nanoparticles that are incorporated on the surface of the material. In such cases, much attention has been given to the synthesis of coatings containing ZnO, SiO_2_, Cu and Ag nanoparticles with a biocidal effect [19,20,21,22]. Although the antibacterial properties of the surfaces by the mentioned procedures have gained more or less success, the cytotoxicity and thus inability to promote adhesion and growth of desired cell types as well as to obtain desired mechanical properties of the coatings, are still not fully solved.

Plasma surface modification techniques are an intriguing way to prepare antibacterial surfaces, not only for better adhesion of antibacterial coatings but also for inducing the formation of antibacterial effects of metal implants. Plasma-assisted surface cleaning may change oxidation, nitration, hydrolyzation, and amination, which increases surface energy and hydrophilicity of the biomaterial [8]. Such changes should reduce the number of attached bacteria and the formation of biofilm.

In present contribution, we discuss different approaches used for surface modification of metals to improve their antibacterial properties. The emphasis is given on the use of plasma technologies for surface modification of implantable materials as one of the compelling ways to alter surface properties in terms of surface chemistry, topography, wettability, surface charge, and crystallinity. All these parameters have a significant impact on the biological response and will be discussed in terms of bacterial adhesion and biofilm formation. The mechanisms of plasma modification of metal surfaces for specific plasma treatment techniques will be presented and critically discussed. Furthermore, this review aims to present the proposed mechanisms of interactions between bacteria and metal biomaterial surfaces in terms of their surface properties; like nanotopography, surface chemistry, crystallinity, wettability, and surface charge. Approaches based on plasma technologies that are already commercially available and the pros and cons of different techniques and their influence on bacterial adhesion, biofilm formation as well as cytotoxicity will be presented.

## 2. Antibacterial Metal Surfaces in Medicine

**Figure 1:**
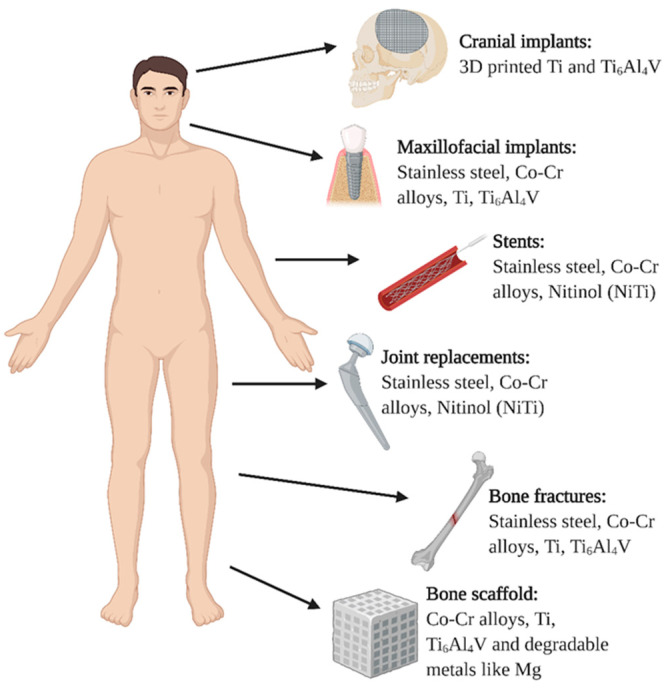
Types of metal implants used in the human body.

The most commonly used biocompatible metal materials for medical tools and implants are titanium and titanium alloys, stainless steels, cobalt-based alloys, magnesium-based alloys and others (tantalum, nitinol, platinum-based alloys, gold alloys, etc.) [4,23,24]. Biocompatible metal materials are frequently used for implantable devices for different applications, as presented in Figure 1. In the case of cranial implants, titanium and its alloys are usually used, where novel 3D printing technologies enabled their vast applications, as implants can be printed according to patient-specific anatomy [25]. Similar goes for the maxillofacial implants where titanium and its alloys, stainless steel, and CoCr are mostly used. Metal implants like titanium alloys, nitinol (NiTi), stainless steel (SS), and cobalt chrome (CoCr) are commonly employed also for vascular implants, like stents. Nitinol is, due to its memory shape properties, a great candidate for vascular implants. However, these implants still lack desired biocompatibility, mostly due to the high risk of stent-induced thrombosis [26,27]. Thus, various surface coating procedures have been developed for these types of implants, not only to prevent bacterial adhesion but also to prevent platelet adhesion and proliferation of smooth muscle cells, while providing sufficient proliferation of endothelial cells. Hence, the mechanisms for the biological response of specific implants differ and the implant surface has to be carefully designed for the given task (implant function). In the case of bone and bone grafts, good osteoblast adhesion has to be considered together with appropriate mechanical and antibacterial properties. For orthopedic implants, titanium and its alloys are considered superior to CoCr and stainless steel due to their lower module of elasticity and better biocompatibility. In the case of stainless steel and CoCr implants the leaching of metallic ions, like Fe, Cr, and Co causes cytotoxic effects to the surrounding cells [28,29,30,31]. While titanium and its alloys show better in vivo biological response, due to the fact that release of Ti ions in the surrounding tissue is less cytotoxic, while the advantage of titanium alloy is also the ability to form a native titanium oxide layer (about 2–10 nm thick) which is biologically inert. Unfortunately, metal surfaces lack desired antibacterial properties therefore significant research efforts have been aimed at modifying surface properties in order to achieve the antibacterial effects.

### 2.1. Antibacterial Strategies

The use of metals, such as silver (Ag), copper (Cu), zinc (Zn) for healing and health benefits stretches back for thousands of years as it was already practiced in ancient times. Romans, for example, have used zinc compounds as the ingredients of drugs used for eye disease treatment [32]. The use of many metals for their antibacterial and biocompatible properties is continued to this day, with several modifications and improvements. The most common field of metal usage in medicine is in the form of medical implants.

Antibacterial properties of metals can be achieved by inserting an antibacterial agent into the material, such as silver (Ag) or copper (Cu), two of several metals that have antibacterial properties [4,8,17,23,33]. Although silver has been used for more than a decade, its toxicity connected issues have limited its use. Besides, it is important to emphasize that introduction of new metals into medical materials can potentially affect the fundamental properties; like stability or processability of the biomaterial (medical device). Surfaces should at the same time also prevent cytotoxicity toward eukaryotic cells and control an undesired inflammatory response. The most commonly employed methods are coating surfaces by plasma immersion ion implantation (PIII), chemical and physical vapor deposition, sol-gel, plasma spraying, plasma electrolytic oxidation (PEO), anodization [34]. The type of surface modification method and biomaterial used depends mainly on its function and position in the body [8].

A variety of metals have toxic effects on bacteria at very low concentrations (nonessential metals) or concentrations above certain threshold values (essential metals) [35]. Metals depicting antibacterial activity are usually found in transition metals (V, Ti, Cr, Co, Ni, Cu, Zn, etc.) or other metals and metalloids (Al, Ga, Ge, etc.). Interestingly, metals have a great toxic effect on bacteria growing as a biofilm and on dormant bacterial cells, which are relatively unaffected by antibiotic treatments [35,36,37]. Based on the antibacterial toxicity mechanisms, metals can be mainly distributed into five groups: protein dysfunction, production of ROS and antioxidant depletion, impaired membrane function, interference with nutrient uptake, and genotoxicity.

An intriguing way to alter the surface morphology and form a biomimetic surface is nanostructuring. The natural or bio-inspired surfaces, like lotus leaf [38], dragon wings [7], gecko [38,39], and shark skin [40] with microstructures and/or dense nanoscale pillars have shown remarkable bactericidal properties [7,38,39], as those nanostructures are able to physically kill adhered bacteria via rupture of a bacterial cell by nanopillar structures. These types of surfaces have shown immense potential in the emerging worldwide epidemic of bacterial resistance to antibiotics as well as hospital-acquired infections, among them also human coronavirus [40].

Additionally, it would be of great interest to design such nanotopographic surface features that would prevent bacterial adhesion and biofilm formation and improve the proliferation of desired cell type. Studies conducted on TiO_2_ nanotubes with different diameters already showed that nanotopography plays an essential role in bacterial adhesion [41], as well as in osteoblast adhesion [42], platelet adhesion, and endothelial cell adhesion [43]. It was shown that alternation in surface features on the nanometer scale significantly reduced bacterial adhesion [44].

Thus, many attempts have been directed at developing surfaces with antibacterial properties, as this would automatically solve the problem of infections caused by implantable devices and hospital-acquired infections. Due to highly complex mechanisms in pathogenesis, these infections have to this date not been well understood. The main issue is the biofilm formation, which significantly differs depending on the affected tissue in contact with the biomedical device and the surface properties of the device.

### 2.2. Bacterial Adhesion and Biofilm Formation on Metal Surfaces

Adhesion of bacteria and biofilm formation on the metal surfaces used in medicine poses a threat of the development of implant-related infections, which remains a leading cause of implant failure, replacement, and chronic diseases [45,46]. Implant-related infections involve interactions between the microbes, the biomaterial, and the host’s immune system [47].

In the presence of physiological fluids, bare metal surfaces are rapidly covered by extracellular matrix (ECM) proteins and immune protein components [48,49]. Protein adsorption is a crucial determinant of cell response to surfaces [50]. However, surface chemistry and wettability control the implant’s coverage by proteins from blood and interstitial fluids, which can happen within nanoseconds [48].

**Figure 2:**
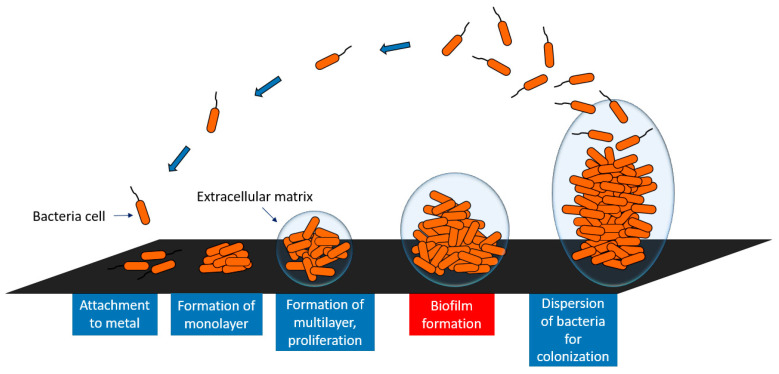
Schematic representation of the biofilm formation on metal surfaces.

The development of a biofilm involves several stages (Figure 2); freely floating bacteria cells adhere to the surface by various weak interactions (Lifshitz–van der Waals, Lewis acid-base, and electrostatic forces) [51,52]. The initial adhesion of microbes depends on the physico-chemical properties of the implant surface [53]. In the next stage, the bacteria irreversibly attach to the surface through strong hydrophilic/hydrophobic interactions and form a monolayer. Further on, bacteria aggregate and produce an extracellular matrix, responsible for supplying nutrients to the bacteria inside the biofilm, adherence between the cells, and mechanical stability of the biofilm [54]. The next step is biofilm maturation and finally, the last stage involves the further dispersion of bacteria as schematically presented in Figure 2.

The bacterial adhesion on the surfaces is also governed by bacterial membrane surface characteristics, such as charge and hydrophobicity/hydrophilicity [55]. The bacterial cell is usually negatively charged, however, the membrane is highly heterogeneous, exhibiting different charges around the cell body [56]. It is believed that negatively charged bacteria easily adhere to surfaces with positive charge [57].

## 3. Plasma Technologies in Medicine

**Figure 3:**
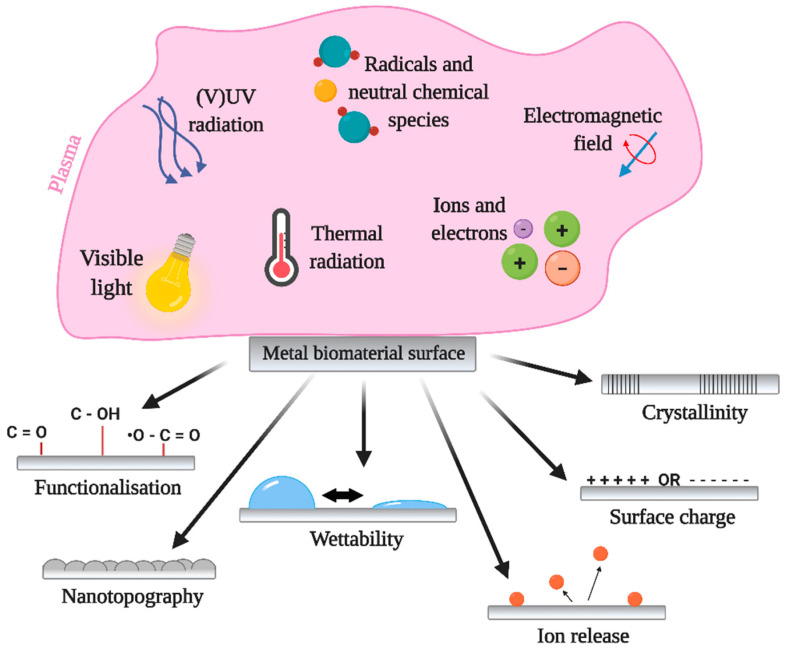
Influence of plasma generated species and its influence on the surface properties of metals used for modification.

Plasma technologies have gained significant importance in the medical field for improving biomaterials’ surface properties. Moreover, the direct use of atmospheric pressure plasmas has also shown great potential in the treatment of chronic wounds or cancer therapy [58] and preventing bacterial adhesion and biofilm formation on different surfaces [59,60]. Plasma is highly efficient in altering the surface properties of biomaterials due to the interaction of highly reactive plasma species with the surfaces. The main advantage of plasma modification is that the top surface layer is modified while preserving the material’s bulk features. Moreover, plasma surface modification is an environment-friendly process, which enables modification of surface chemistry, morphology (on the nanoscale), wettability, surface charge, as well as crystallinity [61], which all influences the biological response. The schematics of plasma interaction with metal biomaterial surface are presented in Figure 3. It is important to note that all mentioned surface parameters play a crucial role in the initial adhesion and growth of bacteria. These surface properties can influence the amount and/or conformation of adsorbed proteins which further dictate bacterial adhesion and biofilm formation. Therefore, the antibacterial surface may be achieved by carefully optimizing plasma treatment conditions in order to obtain desired surface parameters. At this point, it should be also mentioned that plasma coating technologies, where different types of precursors or materials from a second source are used to coat the surface to improve their surface properties are already vastly used in the medical field, especially plasma spraying technology [62,63].

### 3.1. Types of Plasma

Plasma is a word of ancient Greek origins, meaning “moldable substance”. In physics, it describes the fourth state of matter. The sequence in which these states occur is solid, liquid, gas, and then plasma. Plasma is also known as ionized gas and has no shape and no volume (like gas state) but at the same time can be shaped by magnetic fields as it is electrically conductive [64]. The phenomenon of electrical conductivity originates from broken bonds inside gas molecules and atoms resulting in free electrons, charged ions, and other reactive species. According to how these bonds are broken, plasma can be divided into thermal and non-thermal plasma considering the Maxwell–Boltzmann thermodynamic equilibrium [65]. Thermal plasma also known as equilibrium, fully ionized or simply hot plasma, reaches temperatures in the ranges of a tenth of thousands and up to millions of Kelvins making it less applicable as surface treatment processes.

Non-thermal plasmas are generated by exposing gases to non-equilibrium conditions which partially ionize them. Electrical energy is usually used to create these conditions, either directly applying a voltage to the electrode(s) or indirectly by using coils and electric current to generate strong magnetic fields, again ionizing the gas [66]. All of these “artificially” generated non-thermal plasmas can be applied as surface treatments at ambient temperatures (or slightly elevated yet much lower compared to thermal plasma) [67]. Two types of plasma treatment are known in medicine, one is direct where plasma is in direct contact with living cells/tissue/organisms, and indirect where plasma is used to modify materials that are to be later used in living organisms [68,69]. Plasma treatment of metals for improving antibacterial surface properties is considered indirect and is therefore subjected to much less medical scrutiny compared to direct plasma treatments.

When it comes to operating pressure plasma can again be divided into two groups; low-pressure and atmospheric pressure plasma.

#### 3.1.1. Low-Pressure Plasma

**Figure 4:**
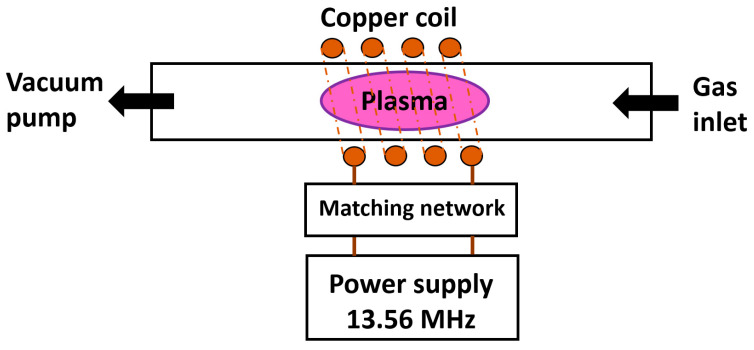
Schematics of ICP low-pressure plasma.

Low-pressure plasma is ignited and sustained inside vacuum vessels (usually made of glass or stainless steel), previously evacuated by vacuum pumps. A schematic representation of a low-pressure plasma system is presented in Figure 4. According to desired effects of plasma treatment, a proper gas is chosen and supplied into the vessel where plasma is sustained. The absence of other gases makes such a process well-controlled, repeatable, and stable. A large area of uniform plasma can be generated, with size and shape depending on the vessel. The most common power sources for generating low-pressure plasma operate on direct current (DC), radio frequency (RF, at 13.56 MHz), and microwave frequency (MW, at 2.45 GHz) discharges. Most configurations allow for two different operating regimes, the so-called E-mode and H-mode where E-mode stands for a lower density, lower temperature, and lower input power compared to H-mode. Plasma discharge can also be ignited locally inside a vessel, for example in an inductively coupled plasma (ICP) configuration presented in Figure 4. Depending on vessel shape and design, samples can be plasma treated in two manners. One is plasma treatment inside glow discharge, where ionized gas species are abundant, and another one is in the so-called after-glow. As the name suggests, after-glow plasma treatment takes place between glow and the vacuum pump, where only long-lived plasma species are still present. Such after-glow plasma treatment allows for surface modification of most delicate materials with great selectivity of plasma species present.

#### 3.1.2. Atmospheric Pressure Plasma

Atmospheric pressure plasma operates, as the name suggests, at atmospheric pressure which usually means that there is no additional vessel and surrounding air present and is also partially ionized [70]. The absence of vacuum pumps and vessels combined with possibilities of in-line industrial applicability made atmospheric pressure plasmas desired. Consequently, much research was performed in recent years and numerous configurations of atmospheric pressure plasmas exist nowadays [66,71]. All of them consist of some kind of electrical power supply and one or more electrodes. Considering the configuration, these atmospheric pressure plasmas can be divided into dielectric barrier discharge (DBD), DBD-like, single electrode jets usually referred to as atmospheric pressure plasma jet (APPJ), and corona discharge. Historically, corona discharge was the first to be invented and is of lesser interest in today’s studies. The single electrode has more potential applications. At the same time, it has its drawbacks such as an ungrounded plasma jet which is highly susceptible to conductive materials and difficulties of evenly treating larger areas [72].

**Figure 5:**
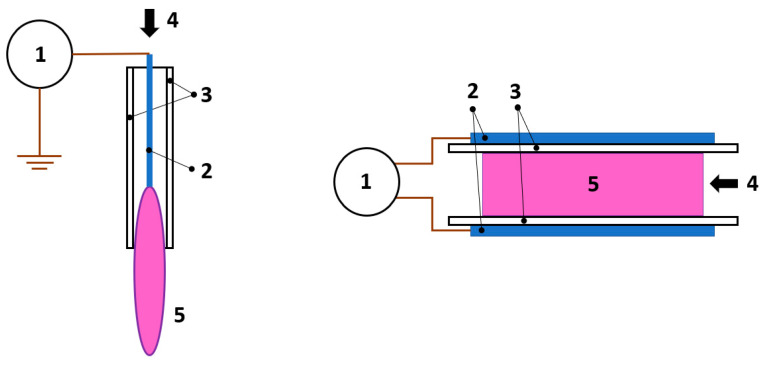
Two schematic configurations of APPJ (left) DBD plasma (right). 1-power supply, 2-electrodes, 3-dielectric barrier, 4-gas flow, 5-plasma.

DBD is the prevailing configuration when it comes to non-thermal atmospheric pressure plasmas [73]. Dielectric materials cover one or both electrodes while high voltages and frequencies in the kHz range are applied to ignite the plasma. Numerous configurations and variations of DBDs exist (and are well described by Lu et al. [74]) whereas it is enough to divide them into two groups for this general review. On the one hand, we have the DBD jets with long cylindrically shaped electrodes or ring electrodes around tubes, inside which the DBD jet is ignited. On the other hand, there are planar DBD configurations with planar electrodes and multiple discharges combined into a large volume plasma. Each configuration has its advantages, but when it comes to preparing antibacterial surfaces, the planar configuration is more efficacious [75]. No matter the configuration, DBDs operate through charge collection on the dielectric layer, resulting in a drop of voltage across the ignited plasma and consequently extinguishing it. Hence DBDs are self-pulsed discharges without the possibility of arching. When ignited, DBDs generate non-uniform plasma composed of many streamers traversing randomly between electrodes. The streamers are short-lived and in the sub-millimeter size range [76]. Uniform diffuse plasma free of streamers can be generated by DBDs, which is of high interest in industrial and biomedical applications [77,78]. Two examples of atmospheric pressure plasma systems, the APPJ (on the left) and DBD (on the right), are schematically presented in Figure 5.

### 3.2. Commonly Employed Plasma Treatment Techniques

**Figure 6:**
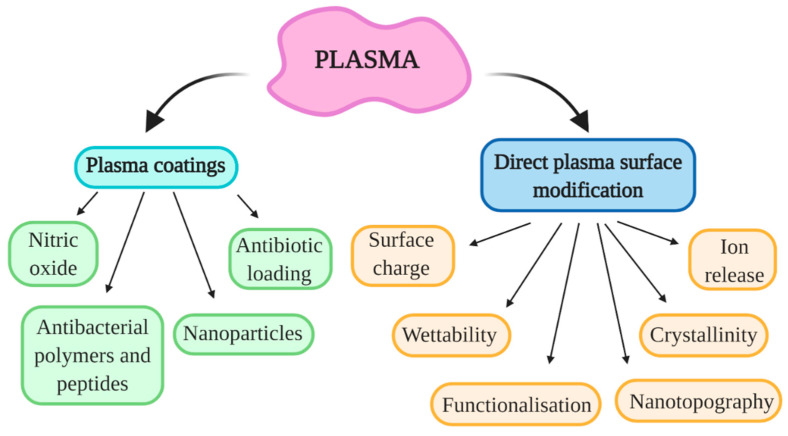
Plasma surface modification.

Plasma is a versatile tool for surface modification of metals and other materials. Not only can it be used directly as a gaseous plasma treatment of surfaces, but it can also be applied in coating processes. The schematic representation of plasma technologies used for deposition and direct gaseous treatment of metal materials is presented in Figure 6. As seen in Figure 6**,** the direct exposure of the metal surface to plasma discharge alters its surface morphology (nano-scale), chemical structure (functionalization), wettability, surface charge, crystallinity, and results in ion release. Thus, properly choosing plasma and discharge parameters, the surface properties may be optimized to obtain antibacterial characteristics [79]. Unfortunately, not all direct gaseous plasma treatment effects are permanent due to surface transiting/recovering back into a more preferable neutral, low-energetic state. It all depends on the substrate material and plasma parameters, but usually, changes in surface morphology and crystallinity are permanent while the other effects fade with time. The time scale of surface recovery is again defined by many parameters and is ranging from seconds after treatment to multiple weeks [80]. By applying coatings, surface recovery is not an issue, thus this type of plasma treatment approach is more commonly used. Nevertheless, with improved knowledge on bacterial reaction to gaseous plasma-treated metallic surfaces and the known window-of-opportunity, the gaseous plasma treatment alone could present great potential for the future. Some of the most common plasma coating techniques are explained and summarized hereon.

#### 3.2.1. Plasma Spraying

**Figure 7:**
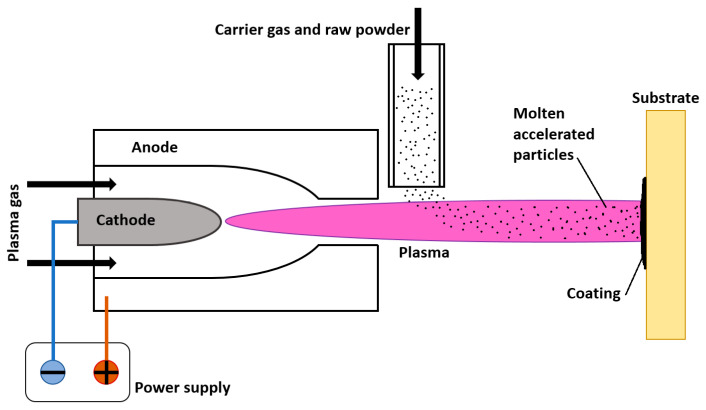
Schematic representation of plasma spraying setup.

Plasma spraying is a thermal spraying process, where finely divided metallic or non-metallic particles are deposited onto the substrate surface in a molten or semi-molten state [81], as schematically presented in Figure 7. The thermal plasma heat source temperatures are between 7000 and 20,000 K, enough to melt any given coating material [15]. The powdered materials are injected into plasma (RF or DC), where they are dissolved and partially melted, landing on the substrate, solidifying and forming splat/lamellae layers of coating. To produce optimal coating, the temperature must be high enough for the coating to reach the substrate in a liquid state (and not resolidify mid-air) yet not too high. In the case of overheating, the particles might decompose or evaporate and never reach the substrate. One of the basic requirements for successful plasma sprayed coating is that the difference between melting and vaporizing/decomposition temperatures of coating is at least 300 K [16], otherwise, coating efficiency drops significantly. Plasma spraying can be performed in air or under a protective atmosphere at different pressures.

The process of plasma spraying can be used to tailor surface characteristics to improve heat, wear, corrosion resistance, and biocompatibility [82,83,84]. It is commonly used to spray high added value coatings. The thickness of plasma sprayed coatings can be as high as 100 µm without any degradation of the substrates’ mechanical properties [85]. Difficulties may occur in complex geometric shapes of substrates as plasma spraying is a line-of-sight process. Plasma spraying technology is commercially used for coating medical implants to improve their surface properties. For example, DOT medical implant solution produces vacuum plasma sprayed (VPS) hydroxyapatite (HA), or titanium plasma sprayed coatings, while HA coatings (commercially Ospravit^®^ surface) by air plasma spray (APS) production process are commercially available from Lincotek Medical. Many other companies produce medical coatings based on this technology, like Dentsply Friadent, Lifecore Dental, Nobel Biocare, etc.

#### 3.2.2. Plasma Induced Physical and Chemical Vapor Deposition

Both physical and chemical vapor deposition can be achieved using plasma as a heat, ionization, or vaporization source. A coating material is vaporized/gasified and deposited onto the substrate under low-pressure or atmospheric conditions in both processes. Physical vapor deposition does not require precursors; once the atoms from the source material are vaporized, they are directly transferred onto the target substrate, where they atomically bond with the substrates’ surface and form a thin layer of coating [86]. This is a line-of-sight process, similar to plasma spraying. The substrate can be rotated to obtain a more consistent coating, but complex geometries pose a serious challenge [87]. Chemical vapor deposition, on the other hand, requires precursors that chemically activate source material vaporized by plasma or low pressure to bond with the substrate via diffusion and form a thin layer of coating [88]. The major advantage of chemical vapor deposition (CVD) compared to physical vapor deposition (PVD) is its ability to form thin layer coatings of consistent thickness over intricate geometries of substrates. Hence, CVD is superior in applying antibacterial coatings [89].

Magnetron sputtering is one of the PVD processes commonly used for thin film deposition. It is conducted in a vacuum chamber in an inert gas atmosphere (preferably high-purity Ar). Magnets are introduced beneath the target material to increase deposition rates, resulting in secondary electrons being bound to the near-surface region of the target. These secondary electrons further improve the intensity of ion bombardment and, therefore, also help to improve the deposition rate of coatings onto the substrate [90].

The TiN, TiNbN, CrN, DLC, and ZrN coatings based on the PVD method are available at DOT medical implants solution, while, for example, Marle orthopedics produces medical coatings based on both the APS and VPS method.

#### 3.2.3. Plasma Electrolytic Oxidation (PEO)

**Figure 8:**
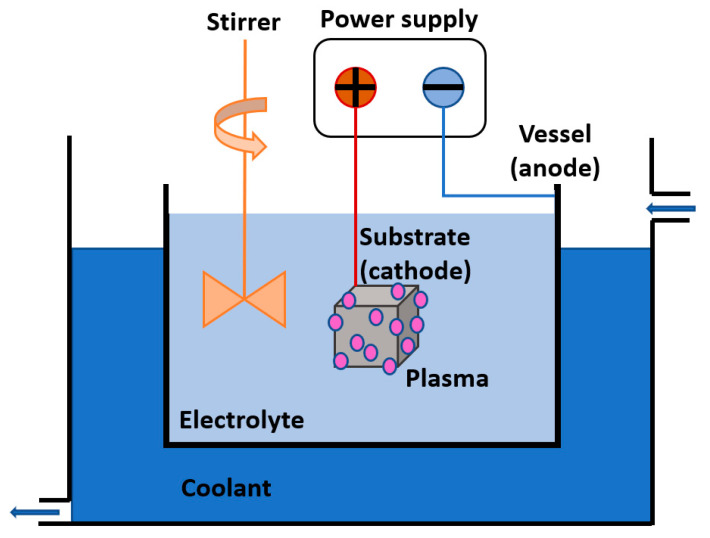
Schematics of PEO.

Plasma electrolytic oxidation (PEO), also called micro-arc oxidation (MAO), anodic spark deposition (ASD), plasma chemical oxidation (PCO), or anodic oxidation by spark discharge (ANOF) [91], is a passivation method for the surface of various metallic substrates in appropriate aqueous electrolytes. Under a strong electric field, a discharge is generated in a system comprising of the substrate submerged into the electrolyte, the cooled vessel, and the power supply, as schematically presented in Figure 8. Plasma is a result of electric break down between the substrate and the vessel, causing anodic polarization and subsequent incorporation of oxygen, alloying elements, and electrolyte components into the substrate. The combination of the substrate (cathode), vessel (anode), electrolyte, distance, temperature, and other parameters play a crucial role in the composition of the produced thin coating and its morphology. Technologically relevant passive films are made by combining electrode metal and electrolyte to exhibit ion conductivity while exhibiting no electron conductivity [90,92,93].

The PEO anodized surface coatings for medical implants were introduced by Nobel Biocare, (commercially TiUnite surface) and Keystone Dental (commercially BioSpark surface).

#### 3.2.4. Plasma Immersion Ion Implantation and Deposition (PIII)

The process is known under many names, but PIII or PIII&D (plasma immersion ion implantation and deposition) are generally used. The “deposition” part stands for applications where thin layers are deposited onto a substrate, in cases where only individual ions are implanted acronym PIII (plasma immersion ion implantation) describes the method. All plasma ion implantation techniques commonly have a high bias applied to the substrate while being immersed or exposed to plasma inside a low-pressure system [94]. Applied substrate bias is usually pulsed to avoid arcing, limit the sheath size, enable new ions to refill near-substrate region, and tailor duty cycles optimally [95]. A high negative voltage applied to the conductive substrate immersed in plasma repels the plasma electrons from the near-substrate region while the ions remain there due to inertia. The ion matrix sheath is formed and after overcoming inertia, the ions are accelerated towards a negatively biased substrate. The force of impact strongly depends on their distance from the surface and the number of collisions along the way. The PIII&D treatment temperatures must stay below critical annealing or phase transition temperatures to preserve the bulk characteristics of the substrate material. The main features of surfaces engineered by PIII&D are superior antibacterial properties, superior adhesion properties, pinhole-free surface films, the formation of metastable phases, and the ability to densely coat complex geometries [96].

Ion doping can be achieved if glow discharge (DC) is applied and plasma is also used for heating the substrate. The result is not only creating surface layers of coating but also the diffusion of ions into the bulk material [97]. The most commonly known types of ion doping are nitriding (N), carburizing (C), nitrocarburizing (N + small amount of C), and carbonitriding (C + small amounts of N). The main application of ion doping is improving wear resistance and thermal fatigue of steels and other ferrous alloys.

## 4. Overview of Plasma Treatment Techniques: Antibacterial Surfaces

The most commonly and commercially employed technique for improving surface properties of medical metal materials is plasma deposition. Plasma deposition of coatings can be applied to various materials, even to heat-sensitive scaffolds and medical fabrics. Plasma technology enables the deposition of thin organic or inorganic films on biocompatible metals for medical implants. The application of antibiotics on medical implants is possible, but with the constant emergence of newly resistant bacteria in hospital infections, more appropriate methods for acquisition of antibacterial surfaces are the incorporation of different compounds or metal nanoparticles on the surface of implants, such as silver, copper, zinc oxide nanoparticles, and others [33,98,99]. Yonezawa et al. [100] used plasma chemical vapor deposition to apply fluorinated diamond-like carbon coating (F-DLC) to a titanium alloy (Ti_6_Al_4_V) commonly used for implants. Coated titanium disks had high antibacterial activity, comparable to already clinically used silver-containing hydroxyapatite (Ag-HA) coating. The presence of fluorine as an antibacterial agent in the coating might be suitable for long-term implants, as there are no reports of fluorine resistant bacteria. Fielding et al. [101] incorporated silver oxide (Ag_2_O) into HA coating on the titanium substrate. Ag_2_O had high antibacterial activity and prevented bacterial colonization on the surface. However, the Ag_2_O also exhibited cytotoxic effects on human fetal osteoblast cells in in vitro study. The addition of strontium oxide (SrO) to the Ag-HA coating alleviated the cytotoxic effects on human cells, but the bacteria’s biocidal effects remained. These studies show that an appropriate combination of biocompatible metals for implants and various coating procedures based on plasma technology present a high potential for the fabrication of antibacterial surfaces used in the field of clinical medicine.

Another intriguing way to gain antibacterial properties of medical implants is nanostructuring of the metal surface, which can also be achieved by plasma treatment. Gajian et al. [102] reported that inductively coupled plasma reactive ion etching under suitable conditions forms titanium nanostructures on titanium surface that simultaneously has biocidal and osteogenic activity. By optimizing the plasma conditions, these effects could be alleviated. Such techniques, where only the nanostructure of the biomedical implant is modified, could have a great potential in the next generation of metal implants, as there are no additional agents added to the material that could affect the overall properties of the medical implant itself.

Compared to other materials titanium and its alloys show great promise for enhancing the functionality of many implantable devices as they possess unique mechanical properties, are anticorrosive and biocompatible due to the formation of protective titanium oxide (TiO_2_) layer. However, the spontaneously formed titanium oxide layer is not uniform and it varies in thickness from 2–10 nm, which influences the biological response (e.g., cells or tissue). Therefore, the characteristics of the titanium oxide layer play a crucial role in the biological response [103,104]. The improved biocompatibility of titanium oxides is believed to be attributed to its surface energy as well as to its n-type semiconductor properties [105]. Therefore, titanium oxide coatings have been deposited on the surface of bulk material in a form of thin-film from a secondary source (like PVD), while a more intriguing way is to form titanium oxide from the existing surface, which eliminates concerns regarding coating instabilities. One of such approaches is oxygen plasma immersion ion implementation (PIII) and non-thermal atmospheric pressure plasma treatment, which were already shown to form titanium oxide or to increase the oxide layer on titanium materials [106,107]. Moreover, plasma treatment is increasingly used in biomedical applications, as plasma-treated surfaces improve biological response. For example, plasma-treated polymeric surfaces were shown to improve the proliferation of endothelial cells and reduce thrombosis [108,109,110] while treatment of Vicriy braided suture caused a significant drop in bacterial attachment [8]. Preliminary results already showed that Ti surfaces treated with radiofrequency (RF) oxygen plasma at high power (1000 W) have antibacterial influence against *Escherichia coli* [8,105]. In the present study, the antibacterial effect was connected with reactive oxygen species formed in the plasma, which altered the TiO_2_ surface layer. By employing plasma treatment to TiO_2_ surfaces, a denser and higher quality oxygen layer could be obtained, moreover, the contaminants like carbon are also removed from the surface [10,111], which influences the biological response. It was also shown that the surface of Ti alloy (Ti_6_Al_4_V) treated with RF oxygen plasma at high power (1000 W) reduces the adhesion of *Staphylococcus aureus* bacteria [111]. In this case, the surface morphology was altered due to ion bombardment and the formation of a thicker oxide layer on the surface. The main problem associated with plasma treatment is the stability of the modification. Usually, the surface tends to return to its more favorable energetic state, via the so-called “ageing” effect, i.e., by reorientation of the newly formed functional groups in the case of the polymeric surface. This is also connected to changes in hydrophilicity, as the formed hydrophilic surface after “ageing” becomes more hydrophobic (hydrophobic recovery). This phenomenon is highly depended on the type of material as well as on the plasma treatment conditions (power, type of gas, treatment time etc.) and it can be observed already a few hours after treatment [112,113]. Due to this drawback, plasma treatment is mainly used as a pre-treatment step that enables better adhesion of various types of coatings [61,114] or as a coating technology where different precursors (CVD, PVD etc.), electrolytes (PEO) or targets (PIII, PVD etc.) are used for deposition.

Plasma nitriding is widely used for surface finishing of stainless steel (SS316), however, it is a time- and energy-consuming process. Only a few reports showed that surface modification of SS316 by efficient but straightforward non-thermal radio frequency (RF) plasma is also feasible; O_2_ plasma treatment resulted in thickening of oxide layer along with oxidation of the surface species–Cr, Fe and Mo to their highest valence states and segregation of Ni away from the surface.

The low-pressure plasmas are more efficient in surface modification compared to atmospheric pressure ones, however, in recent years, the interactions between non-equilibrium (cold) atmospheric pressure plasmas and liquids attracted significant attention due to their applications in medicine and nanomaterials synthesis. The PEO techniques enable the introduction of versatile inorganic metal elements (e.g., Ag, Cu, Zn) into the coating, which could significantly improve antibacterial surface properties. However, it is necessary to precisely control the concentration and content of the antibacterial metal elements, electrolytes as well as PEO parameters to optimize antibacterial properties and at the same time prevent cytotoxicity. It is also possible to enhance both the antibacterial properties as well as bioactivity by the simultaneous addition of antibacterial metal elements and bioactive elements, such as strontium (Sr) and silicon (Si) in the PEO electrolyte [90]. For instance, Santos-Coquillat et al. [115] treated Ti_6_Al_4_V alloy with PEO using an F-containing electrolyte. The authors observed a significant reduction of bacteria adhesion (*Staphylococcus aureus*) on the surface with fluoride’s highest content.

In comparison to untreated Ti_6_Al_4_V alloy, the plasma modified developed thick biofilm [115]. The incorporation of metal nanoparticles into the coatings generated by PEO also improves the surface’s antibacterial properties. For instance, Ag nanoparticles-decorated TiO_2_-based coatings on titanium exhibited bactericidal effects against *E. coli* and *S. aureus* bacteria strains [116]. Zhou et al. [117] linked the antibacterial mechanism (*E. coli* and *S. aureus*) of the W-containing coating on the surface of Ti_6_Al_4_V alloy formed by PEO with the reactive oxygen species (ROS). The proposed antibacterial mechanism is as follows; ROS formed by nano-sized W, attack the constituents of the bacterial membrane (e.g., lipopolysaccharide, phospholipid, lipoprotein) and cause the deleterious change in peptidoglycan membrane of *E. coli* (Gram-negative), while ROS directly attack the peptidoglycan membrane and interact with the teichoic acid of *S. aureus* (Gram-positive). The interaction between nanoparticles and gram-negative/gram-positive bacteria is, however, different. The gram-positive bacteria consist of a thick peptidoglycan layer that acts as a protective layer, while gram-negative bacteria lack such a barrier and nanoparticles can easily penetrate the membrane. Gram-negative bacteria also easily react with nanoparticles due to surface charge affinity [34].

These types of coating procedures have recently gained attention and many studies on this topic and some procedures are already commercially available for coating of medical devices. In Table 1, examples of different plasma treatment techniques used for antibacterial surface properties of different materials are presented.

## 5. Plasma induced Antibacterial Properties of Metals

The effect of plasma treatments on metal surfaces and their antibacterial properties can be highly synergistic. Plasma treatments can induce the formation of oxides, induce changes in nanotopography and crystal structure, alter surface chemistry, wettability, surface charge and functionalize the surface. In the following sections, the most significant effects of plasma treatment on the surface of metals are presented and the mechanisms that prevent biofilm formation are discussed.

### 5.1. Influence of Surface Chemistry, Crystallinity, and Ion Release

Plasma treatment can induce the formation of the oxide layer on the surface of metals [129] and can also cause crystallization of amorphous oxide [130]. Surface chemical composition, as well as surface crystallinity, may significantly influence the biological response. It was shown that altering the surface crystalline structure of the titanium oxide layer improved their antibacterial properties. Crystalline anatase phase titanium oxide layer was shown to significantly reduce bacterial attachment in *Streptococcus* bacteria (*S. mutans, S. salivarius, S. sanguis*). Moreover, a crystalline anatase enriched layer improves antibacterial properties without negatively affecting the cell metabolic activity [131]. Titanium oxide antibacterial properties may also arise from its photocatalytic activity based on its n-type semiconducting nature [132] which is also correlated with its crystalline structure. If titanium oxide surface is photo-irradiated with photon energies larger than the band gap it catalyzes oxidation/reduction reactions. This is due to the production of electron-hole pairs in the oxide layer, which are produced by the transfer of a valance band electron to the conduction band. The anatase crystalline structure has a larger band gap (3.23 eV) compared to the rutile crystalline structure (3.02 eV), which increases its surface redox potential [133]. In the case of the biological environment, the valance band oxidizes water molecules to form hydroxyl radicals which plays a pivotal role in cell interaction. It is well known that reactive oxygen species (ROS) like hydrogen peroxide and hydroxyl radicals or superoxide anion can cause oxidative damage to the cell membrane. The disrupted cell membrane does not have sufficient ability to control substances’ movement through the bacterial wall, which eventually causes cell death [134]. Additionally, this type of UV irradiation can have a more pronounced effect on gram-negative bacteria, which have a thinner cell wall compared to a thicker one of the gram-positive bacteria [135,136,137].

Moreover, irradiation with ultraviolet (UV) light also increases the surface wettability of titanium dioxide [80,138] mainly due to an increase in surface hydroxyl groups [139], which again influences bacterial adhesion. In vitro results show that Ti_6_Al_4_V surface exposed to UV inhibits bacterial adhesion without compromising the desired response of osteoblast cells [131], while the similar antibacterial activity of UV irradiated titanium oxide surfaces was observed for *E. coli, S. aureus, P. putida* and *L. innocua* by Bonetta et al. [140]. Moreover, in vitro and in vivo experiments showed that UV-treated titanium substrate substantially enhances its osteoconductive capacity, mainly attributed to its photocatalytic activity [141]. Our previous studies, conducted on oxygen plasma treated TiO_2_ nanotubes showed increased wettability, crystallinity and higher oxygen content on the surface which was correlated with improved in vitro biological response (proliferation of osteoblast cells and endothelial cells) [80,130]. These effects could be attributed to the photocatalytic activity of the titanium oxide layer induced by vacuum ultraviolet radiation (VUV) and ion bombardment, as well as the removal of hydrocarbon contamination. In another study atmospheric plasma jet (APPJ) was used as a source of UV light and reactive oxygen species which inhibited the growth of both gram-negative and gram-positive bacteria, although a more pronounced decrease was observed in gram-negative bacteria, probably due to thinner cell wall. Additionally, such plasma treatment also reduced biofilm formation rate in similar manner (lower biofilm formation for gram-negative bacteria) [59]. Nevertheless, it should be emphasized that TiO_2_ wide band gap semiconductor could also be modified by ions from plasma, such as COOH^−^, NO^−^, OH^−^, N^3−^, and O^2−^, however this type of treatment has only a temporary effect on bacterial adhesion, as the ions only remain on the surface for a limited period of time [142].

The release of bactericidal ions from the surface of biomaterial is another approach to prevent bacterial adhesion. Ion release presents one of the most important factors affecting cell/material interaction and biomineralization. Ions can inhibit the bacteria by damaging their envelope and cytoplasmic component, blocking the peptidoglycan ability to transfer oxygen, inactivation of enzymatic functions of proteins, and/or disrupting the DNA replication [116]. In this case, the most efficient plasma treatment techniques are based on coating technologies (e.g., PIII&D and PEO).

### 5.2. Influence of Nanotopography

Surface nanostructuring of metal implants is used to increase the resistance to infection without the use of antibiotics. It has been shown that some types of bacteria (*E. coli, P. aeruginosa, S. aureus and S. epidermidis*) experienced lower adhesion to the surface with patterning smaller than the size of the bacteria cell [143,144,145].

Plasma treatment can induce the formation of nanotopography on metal surfaces [130,146,147]. Vassallo et al. [148] showed that nanoprotrusions on the surfaces of Si fabricated by plasma treatment method using a tetrafluoromethane (CF_4_) and hydrogen (H_2_) mixture generate produce a mechanical bactericidal effect against three different microorganisms: gram-negative (*Escherichia coli*), gram-positive (*S. aureus*) and spore-forming bacteria (*B.cereus*). Veerachamy et al. [149] prepared nanostructured Al-Ti coating on the Ti_6_Al_4_V by plasma spraying and showed that nanotopography improved antibacterial activity.

It is essential to separate the effects of surface chemistry from those of nanopatterns. In a biophysical model, the cell lysis caused by the bacterial membrane’s rupturing arises from the penetration of high aspect ratio nano-features [150]. This model was confirmed by the study of Elbourne et al. [151], in which authors showed that the bactericidal activity of the gold nanospikes is physical in nature. Nanostructured surfaces or biomimetic surfaces that enable the killing of bacteria by contact have generated significant scientific attention. A report from Ivanova and co-workers confirmed this mechanism; the authors showed that surface nanopillars resembling dragon-fly surface architecture can kill medically relevant pathogens with excellent efficiency [152].

### 5.3. Influence of Surface Charge and Wettability

The bacteria’s attachment to the implant depends on the surface characteristics, mainly surface charge and hydrophobicity [153]. Plasma treatment can alter the surface charge and consequently wettability of metal surfaces [154]. Lee et al. [59] presented the reduced adhesion and biofilm formation on the Ti surfaces treated by non-thermal atmospheric pressure plasma. Authors demonstrated that bactericidal effect arises from the changes in titanium surface properties, such as surface energy, chemical composition, and reductive potential induced by plasma treatment, since no differences in the samples’ topography were observed. Huang et al. [155] showed that increasing nitrogen-content in the N-PIII-treated Ti_6_Al_4_V surfaces decreased the adhesion of gram-positive dental bacteria (*S. salivarius*). Authors proposed that the negative charge of the N-PIII-treated Ti_6_Al_4_V surface acts repulsively towards the negatively charged membrane of *S. salivarius*.

However, the cell envelope’s composition (gram-positive, gram-negative) affect bacteria adhesion to surfaces. As a general rule, the negatively charged bacteria more readily colonize the surfaces with a positive charge and vice versa [57]. Moreover, the bacteria with the membrane’s hydrophobic nature readily colonize hydrophobic materials and vice versa [57].

## 6. Influence of Gaseous Plasma Treatment on Surface Properties

### 6.1. Surface Chemistry

Radiofrequency oxygen plasma was used to alter the surface properties of different types of metals. Results of surface chemistry before and after plasma treatment obtained from X-ray Photoelectron Spectroscopy (XPS), which is a highly surface-sensitive technique, are presented in Table 2. It can be observed that plasma treatment of Ti, Ti_6_Al_4_V, and NiTi removes carbon contamination on the surface and increases the concentration of oxygen. It can be observed from Table 2 that initially all surfaces have almost 70 at% of carbon on the surface, which is due to contamination of the surface with carbon. Immediately after oxygen plasma treatment a significant decrease in carbon and an increase in oxygen is observed on all modified surfaces. It should be mentioned that the NiTi sample was subjected first to H-mode plasma in order to remove carbon contamination and reduce surface oxides, followed by oxygen plasma for preferential formation of titanium oxides. Thus, a much lower concentration of carbon after plasma treatment of NiTi observed in Table 2 could also be attributed to surface pre-treatment by hydrogen. An increase in Ti was observed for all surfaces, interestingly in the case of the NiTi sample, no Ni was detected on the surface not before not after plasma treatment, which confirms that the top surface layer is covered with titanium oxide (in the case of untreated sample native titanium oxide layer which is about 2–10 nm thick). In the case of Ti_6_Al_4_V, a small amount of Al, as well as V, was detected on the surface, which increased after plasma treatment, indicating an increase in aluminum and vanadium oxides on the surface. These results indicate that oxygen plasma treatment indeed increases the titanium oxide layer on the surface of Ti and NiTi, which was shown to beneficially influence the biological response, while in the case of Ti_6_Al_4_V also aluminum and vanadium oxides were formed on the surface.

### 6.2. Surface Wettability

**Figure 9:**
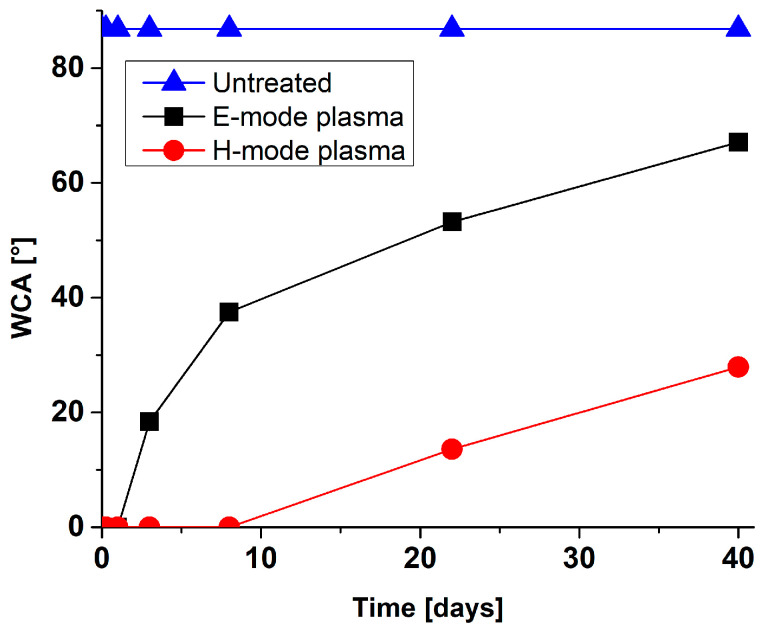
Water contact angles (WCA) for plasma-treated Ti_6_Al_4_V measured for a 40-day period.

**Figure 10:**
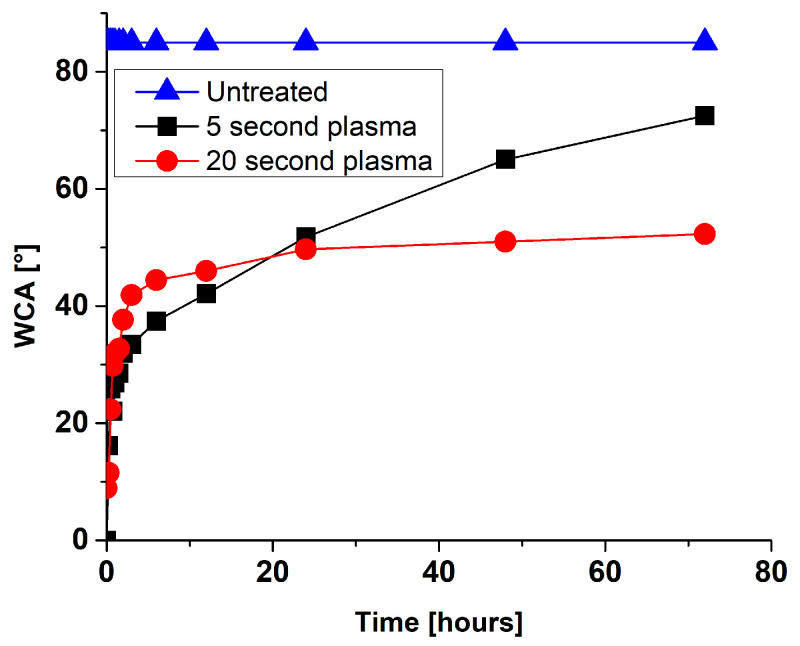
Water contact angles (WCA) for plasma-treated NiTi measured for a 3-day period.

Gaseous oxygen plasma treatment of different metals was shown to render surface hydrophobic character to a hydrophilic one. This is mainly due to the removal of hydrocarbon contaminants from the surface and the formation of hydroxyl groups on the top surface. However, metals’ surface properties are not stable with time, which results in the increased water contact angle (increased hydrophobicity). The so-called “ageing” effect of plasma-treated surfaces is observed, which is in the case of metal surfaces partially attributed to rapid contamination of surfaces with carbon when exposed to the atmosphere. Significant slower ageing was for example observed in the case of metals stored in sealed containers. Moreover, it is essential to emphasize that hydrophobic recovery depends on the type of metal surface and the plasma treatment condition (like plasma power input, type of gas used for modification, treatment time, etc.) In Figure 9 and Figure 10. example of surface wettability after gaseous oxygen plasma of Ti_6_Al_4_V and NiTi surface is presented, respectively. Two different treatment conditions were used for Ti_6_Al_4_V as well as NiTi surfaces. In the case of Ti_6_Al_4_V, predominantly plasma input power was varied (200 W for the E-mode plasma treatment and 600 W for the H-mode plasma treatment). It can be seen that with higher power input surface ageing is significantly reduced. After 40 days of ageing, the E-mode plasma-treated surfaces displayed a drastic change in hydrophilicity. The water contact angle (WCA) after plasma-treatment was below 5° and increased to about 70° after 40 days, which was still below the initially measured WCA of the untreated surface (86.4°). In the case of H-mode plasma-treated surface, the WCA raised to only about 30° after 40 days of ageing. To some extent, this could be correlated also with changes in surface morphology, which were more prominent after treatment of surfaces at higher plasma power inputs (H-mode) as well as to altered chemical composition of titanium oxide layer, even altered crystallinity. In the case of NiTi metal surfaces, a power input of 200 W was used, but treatment time was different (5 s or 20 s). Results presented in Figure 9 show that longer plasma treatment significantly influences the NiTi surface’s hydrophobic recovery. Already after three days of ageing, the 5 s treated NiTi surface reached WCA of about 75°, which is close to the WCA of the untreated surface (85.1°). While in the case of 20 s plasma treatment the WCA reached about 50° in 3 days. In this case differences in nanotopography were observed due to longer treatment time as more pronounced nanostructures were observed on this type of surface. It is also interesting to observe the time of ageing for two types of metals (Ti_6_Al_4_V and NiTi), as NiTi surface showed a significant change in wettability immediately after plasma treatment and reached the initial value already after three days of ageing (for 5 s treated surface). On the contrary, the Ti_6_Al_4_V surface showed a completely different ageing regime, as even after 40 days the WCA was below the initial value. At this point, it should be mentioned that the plasma power input used for the treatment of NiTi samples was 200 W and could to some extent be compared to Ti_6_Al_4_V treatment in E-mode. It should be added that this type of information is crucial for the evaluation of biological response as well as for further processing of plasma-treated materials.

### 6.3. Surface Nanostructuring

Modification of surface by highly reactive oxygen plasma may induce changes in surface morphology, this is commonly observed for polymeric materials due to the preferential etching of polymer matrix, while nanostructuring may be achieved in the case of metal materials, especially at higher plasma power inputs or at longer exposure times e.g., by reactive ion etching. The example of altered surface morphology of Ti, Ti_6_Al_4_V, and NiTi is presented in Table 3. The AFM and SEM images show that the untreated surfaces have no special morphological features, while treatment at specific plasma conditions results in the formation of nano-cones, which were already shown to influence the biological response in terms of proliferation of human cells and bacterial adhesion. The appropriate nanostructured surfaces could at the same time prevent bacterial adhesion and promote the growth of desired cell type.

## 7. Conclusions

Bacterial infection still presents a serious threat to human life mainly due to many antibiotic-resistant bacterial strains. In recent years the medical community and medical device industry have given high recognition to the problem associated with bacterial infections. This has driven many innovative approaches, where plasma-based technologies have shown high potential. New technologies, like 3D printing, have opened new possibilities in designing medical implants. Thus, the design of many implantable devices has significantly changed over the years, while the medical implant-connected bacterial infections have practically remained the same. The revision surgeries due to implant infections still present a serious concern and these issues were not yet properly solved. In this review, we present plasma-based surface treatment techniques, some of them are already used by the medical device industry, while some are in the initial stage of research and/or development. The most commonly employed plasma treatment techniques are based on plasma coating technologies; however, the great potential of direct gaseous plasma treatment of metal surfaces is also foreseen. Approaches based on biomimetic surfaces show great potential, as surface-specific features on the nanometer scale were shown to prevent bacterial adhesion while promoting adhesion and proliferation of desired cell type and thus hold great promise for the future.

## Figures and Tables

**Table 1:** The plasma treatment techniques, used for providing antibacterial surface properties of various materials.

Author	Plasma Treatment Technique	Material	Bacteria	Effect
Miola et al. [118]	Plasma spray	Silver doped glass-coating on Ti alloy	*Staphylococcus Aureus*	The coating induced antibacterial activity. The surface gained bacteriostatic properties without any cytotoxicity.
Brohede et al. [119]	PVD	Bioactive TiO_2_ anatase with hydroxyapatite (HA) loaded with an antibiotic (Amoxicillin, Gentamicin sulfate, Tobramycin, and Cephalothin)	*Staphylococcus Aureus*	After 24 the drug release was high enough to achieve bacterial inhibition. Longer loading times do not mean higher drug release.
Kang et al. [120]	PVD	TiAgN thin film on Ti	*Streptococcus mutans*	Antibacterial properties of TiAgN coating were evident at 5 wt% Ag concentration. No cytotoxic effect on (human gingival fibroblast (HGF) cells was detected. The proliferation of HGF cells even increased after exposure to various AG content on TiAg alloy.
Cao et al. [18]	Plasma spray/PIII	Ag nano particles (NP) 5–25 nm incorporated into titanium oxide coating	*Streptococcus aureus, Echerichia coli*	The size of Ag NP influenced the biocidal activity. Larger particles (5–25 nm) stimulate tougher oxidation reactions than smaller ones (~4 nm). No cytotoxic effect of Ag NP on osteoblast-like cells from mouse (MG63 and MC3T3 cell lines), the cytocompatibility was improved.
Fielding et al. [101]	Plasma spray	Pure titanium coated with HA with silver and strontium 155	*Pseudomonas aeruginosa*	Improved antibacterial activity against *P. aeruginosa* and increased viability, proliferation, and differentiation of human osteoblast cells (hFOB), compared to only HA-coated samples.
Chen et al. [121]	Plasma spray	Titanium substrate coated with HA with silver 158	*Escherichia coli, Pseudomonas aeruginosa, and Staphylococcus aureus*	The HA-Ag coating exhibited high antibacterial activity against all three bacteria. The in vitro study on fibroblast cell line L929 showed no cytotoxicity or hemolytic characteristics for the HA-Ag coating
Yoshinari et al. [122]	PIII and deposition 162	F^+^ ion implementation	*Porphyromonas gingivalis, Actinobacillus actinomycetemcomitans*	F^+^ implanted samples exhibited significant inhibition of bacterial growth for both bacteria. Other surface modified samples did not exhibit antibacterial activity. The F^+^ implanted samples did not inhibit the proliferation of fibroblast cells (L929 cell line)
Xu et al. [123]	PIII and deposition	Zn ion deposition on titanium surface	*Streptococcus mutans*	With increasing Zn concentration on the titanium surface, the bacterial adhesion on samples decreased
Xu et al. [124]	CVD 167	Graphitic C_3_N_4_ on aligned TiO_2_ nanotube layer	*Escherichia coli*	Graphitic C_3_N_4_ composite showed bactericidal properties under visible-light-induced photocatalytic formation of reactive oxidative species.
Gu et al. [125]	CVD 176	Single-layer graphene sheets onto titanium discs	*Staphylococcus aureus, Escherichia coli*	Graphene coating of titanium discs improved cell adhesion and osteogenic differentiation of hASC, hGF and hBMMSC cell lines. Graphene surface showed antibacterial properties on both *E. Coli* and *S. aureus* bacteria.
Cerchier et al. [126]	PEO	Ag particles onto Al surface in an electrolyte	*Staphylococcus aureus, Escherichia coli*	Silver NP on the substrate depicted antibacterial activity against both *E. coli* and *S. aureus*.
Karabudak et al. [127]	MS and MAO	Ag/TO_2_ and Ag NP/TiO_2_ onto NiTi	*Staphylococcus aureus, Pseudomonas aeruginosa, Listeria monocytogenes*, *Escherichia coli*, *Yersinia enterocolitica, Salmonella Enteritidis, Bacillis subtilis*	Samples with Ag NP TiO_2_ showed antibacterial activity on *S. aureus, P. aeruginosa, L. monocytogenes, E. coli* bacteria, which are classified as moderately sensitive, while *Y. enterocolitica, S. Enteritidis, B. subtilis* were classified as resistant to the antibacterial coating. Non-coated NiTi surface was found with the best antibacterial activity for all bacteria.
Jin, et al. [13]	PVD	Cu-Ti ions coated onto 316L stainless steel	*Escherichia coli*	The Cu-Ti coating exhibited great antibacterial activity with an effective reduction of 99.9% of *E. coli* bacteria in the first 12 h. The authors predict that the release of Cu ions has bactericidal properties on *E. coli.*
Li et al. [128]	Plasma spray	Ag nanoparticles onto Ti_6_Al_4_V	*Staphylococcus aureus, Escherichia coli*	Ag coated surface exhibited excellent antibacterial activity.
Lee et al. [59]	APPJ Direct plasma treatment of bacteria	Titanium surface	*Streptococcus. mutans*, *Staphylococcus. aureus*, *Klebsiella oxytoca* and *Klebsiella pneumoniae* on NTAPPJ treated titanium	Lower adhesion of bacteria and biofilm formation rate compared to untreated samples. The adhesion of cells and biofilm formation rate of gram-negative bacteria was significantly lower than gram-positive bacteria.

**Table 2:** Surface chemical composition of Ti, Ti_6_Al_4_V and NiTi before and after plasma treatment (+ P) obtained from XPS analysis.

Material	Concentration at%					
	C	O	Ti	N	Al	V
Ti	68.4	26.1	4.6	0.9	/	/
Ti + P	36.4	51.1	12.5	/	/	/
Ti_6_Al_4_V	69.1	27.2	2.9	/	0.7	0.1
Ti_6_Al_4_V + P	43.8	42.3	11.4	/	2.3	0.2
NiTi	71.7	25.0	2.0	1.3	/	/
NiTi + P	21.0	50.5	28.5	/	/	/

**Table 3:** Nanostructures obtained on plasma-treated samples compared to the untreated ones. The white bar on SEM images represents 1 µm.

	Untreated	Plasma Treated
Ti_6_Al_4_V (AFM)	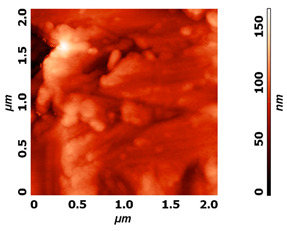	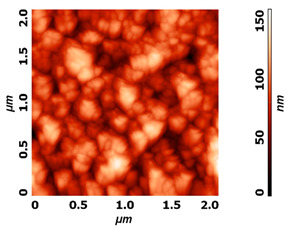
NiTi (AFM)	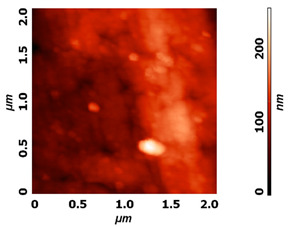	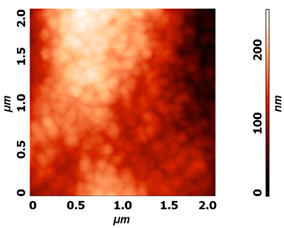
Ti (SEM)	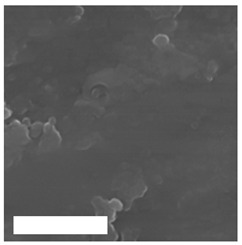	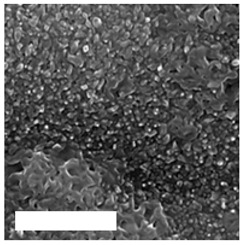

## Data Availability

Not applicable.
